# Patient‐reported outcomes on empagliflozin treatment in glycogen storage disease type Ib: An international questionnaire study

**DOI:** 10.1002/jmd2.12364

**Published:** 2023-04-19

**Authors:** Sarah C. Grünert, Annieke Venema, Jamas LaFreniere, Blair Schneider, Enrique Contreras, Saskia B. Wortmann, Terry G. J. Derks

**Affiliations:** ^1^ Department of General Pediatrics, Adolescent Medicine and Neonatology, Faculty of Medicine Medical Center‐University of Freiburg Freiburg Germany; ^2^ Division of Metabolic Diseases, Beatrix Children's Hospital, University of Groningen University Medical Center Groningen Groningen The Netherlands; ^3^ Sophie's Hope Foundation (CureGSD1b) Hopkinton Massachusetts USA; ^4^ Asociacion Española de Enfermos de Glucogenosis (Spanish Patient Organisation for Glycogen Storage Diseases) Santiago de Compostela Spain; ^5^ University Children's Hospital Salzburg, Paracelsus Medical University Salzburg Austria; ^6^ Amalia Children's Hospital, Radboudumc Nijmegen The Netherlands

**Keywords:** empagliflozin, glycogen storage disease type Ib, neutropenia, quality of life, SGLT2 inhibitor

## Abstract

In patients with glycogen storage disease type Ib (GSD Ib), quality of life is severely hampered by neutropenia and neutropenia‐associated symptoms. SGLT2 inhibitors are a new treatment option and have shown improved medical outcomes in more than 120 patients so far. The aim of this international questionnaire study was to assess patient‐reported outcomes of this new treatment in GSD Ib patients. Patients and caregivers of pediatric patients were invited to complete a web‐based questionnaire. This was designed to evaluate treatment effects of the SGLT2 inhibitor empagliflozin on clinical symptoms and important aspects of daily life including physical performance, sleep, social and work life, traveling, socioeconomic aspects, and quality of life. The questionnaire was completed by 73 respondents from 17 different countries. The mean duration of treatment was 15 months, the cumulative treatment time was 94.8 years. More than 80% of patients reported an improved quality of life. The number of hospitalizations was reduced (66% of patients), as well as the number of days absent from school or work. Granulocyte colony‐stimulating factor (G‐CSF) treatment could be stopped in 49% of patients and reduced in another 42%. Clear improvement of neutropenia and all neutropenia‐associated symptoms was reported by the majority of patients. Additionally, patients or caregivers reported positive effects on appetite (63%), level of activity (75%), overall well‐being (96%), and sleep (63%). Empagliflozin positively impacts many aspects of daily life including work and social life and thereby significantly improves quality of life of patients and caregivers.

**Synopsis**

Most patients with GSD Ib benefit from empagliflozin treatment, and this new therapeutic option significantly improves quality of life of patients and caregivers.

## 1 INTRODUCTION

Glycogen storage disease type Ib (GSD Ib) is an ultra‐rare inborn error of glycogen metabolism due to biallelic pathogenic variants in *SLC37A4*.^1^ The disorder is clinically characterized by recurrent fasting hypoglycemia, hepatomegaly, neutropenia, and neutrophil dysfunction, associated with inflammatory bowel disease, oral and anogenital mucosal lesions, recurrent skin infections and anemia.^1^ Treatment with granulocyte colony‐stimulating factor (G‐CSF) has been the mainstay of therapy for neutropenia in GSD Ib patients since the 1990s.^2^ However, G‐CSF does not address neutrophil dysfunction but solely increases the number of dysfunctional neutrophils and, therefore, often had limited effects. Since the elucidation of the mechanism of neutrophil dysfunction in GSD Ib as a metabolite repair defect in 2019, SGLT2 inhibitors have recently emerged as a new therapeutic option.^3^ Within the last few years, an increasing number of GSD Ib patients have received the counter‐intuitive but successful treatment with the anti‐diabetic drug empagliflozin as off‐label treatment. We have recently published on its safety and efficacy in 112 GSD Ib patients.^4^ The results showed that empagliflozin had positive effects on biomedical outcomes related to neutropenia and neutrophil dysfunction. The majority of patients were able to either stop G‐CSF treatment or significantly reduce the dose.

The use of SGLT2 inhibitors is a breakthrough in the therapy of GSD Ib, and empagliflozin has the potential to become the first‐line treatment of neutropenia and neutrophil dysfunction in this disorder. The aim of this questionnaire study was to assess the patient‐reported outcomes (PROs) and to explore the impact of empagliflozin treatment on GSD Ib patients' quality of life.

## 2 MATERIALS AND METHODS

A web‐based English questionnaire (Survey Monkey) was designed to address the impact of treatment with empagliflozin on clinical symptoms but also other important aspects of daily life including physical performance, sleep, social and work life, traveling, socioeconomic aspects, and quality of life (QoL; Supplemental Material S1). The questionnaire was developed by doctors in collaboration with patients and patient representatives. Patients or caregivers of pediatric patients were invited to participate either by their metabolic physicians or by national patient organization for GSDs. All data were analyzed with descriptive statistics. For this retrospective non‐interventional study collecting anonymized data, no ethics approval was necessary.

## 3 RESULTS

The questionnaire was completed by 73 respondents from 17 different countries. Respondents comprised 27 (37%) GSD Ib patients, 37 (51%) mothers of a GSD Ib patient, 6 (8%) fathers of a GSD Ib patient, 1 (1%) son of a GSD Ib patient, and 2 questionnaires were filled in by the families together with their treating physician who helped with the translation of the survey for these non‐English‐speaking families. The median age of patients at time of the survey was 11 years (range 0–48 years). Empagliflozin treatment was started at a median age of 10 years (range 0–47 years). In three patients, empagliflozin treatment was initiated within the first year of life. The mean duration of treatment was 15 months (range 2–36 months). The cumulative treatment time was 94.8 years. The daily dose ranged between 0.05 and 1.9 mg/kg/day with a median dose of 0.36 mg/kg/day. In the majority of patients, the daily dose was split in two (*n* = 48, 66%), while the remainder received one single dose of empagliflozin per day (*n* = 25, 34%). Treatment costs were covered by the health insurance or the national health system in most countries. However, 22% of patients/families had to pay (fully or in part) for the medication themselves.

Ninety percent of patients (66/73) reported to have received G‐CSF treatment before empagliflozin was started. In 35 of 71 patients (49%), G‐CSF treatment was stopped after the introduction of empagliflozin, and in 30 of 71 patients (42%), either the dose, the frequency of injections or both could be reduced. In 6 of 71 patients (7%), no tapering of the G‐CSF dose was possible or has been tried. (Two patients who reported to use G‐CSF did not answer the questions on follow‐up). The burden of daily injections is perceived very differently among patients/caregivers. When asked to rank how difficult regular G‐CSF injections were for the patient on a scale from 1 (not difficult at all) to 7 (very difficult), responses were evenly distributed across the spectrum with a median rank of 4. Nevertheless, patients who could already stop G‐CSF or reduce the dose/frequency of dosing perceived this as a great relief (*n* = 59; scale from 1 to 7 with 1 = great relief, 7 = no relief; mean rank 2.6, median rank 1.0, range 1–6).

**FIGURE 1:**
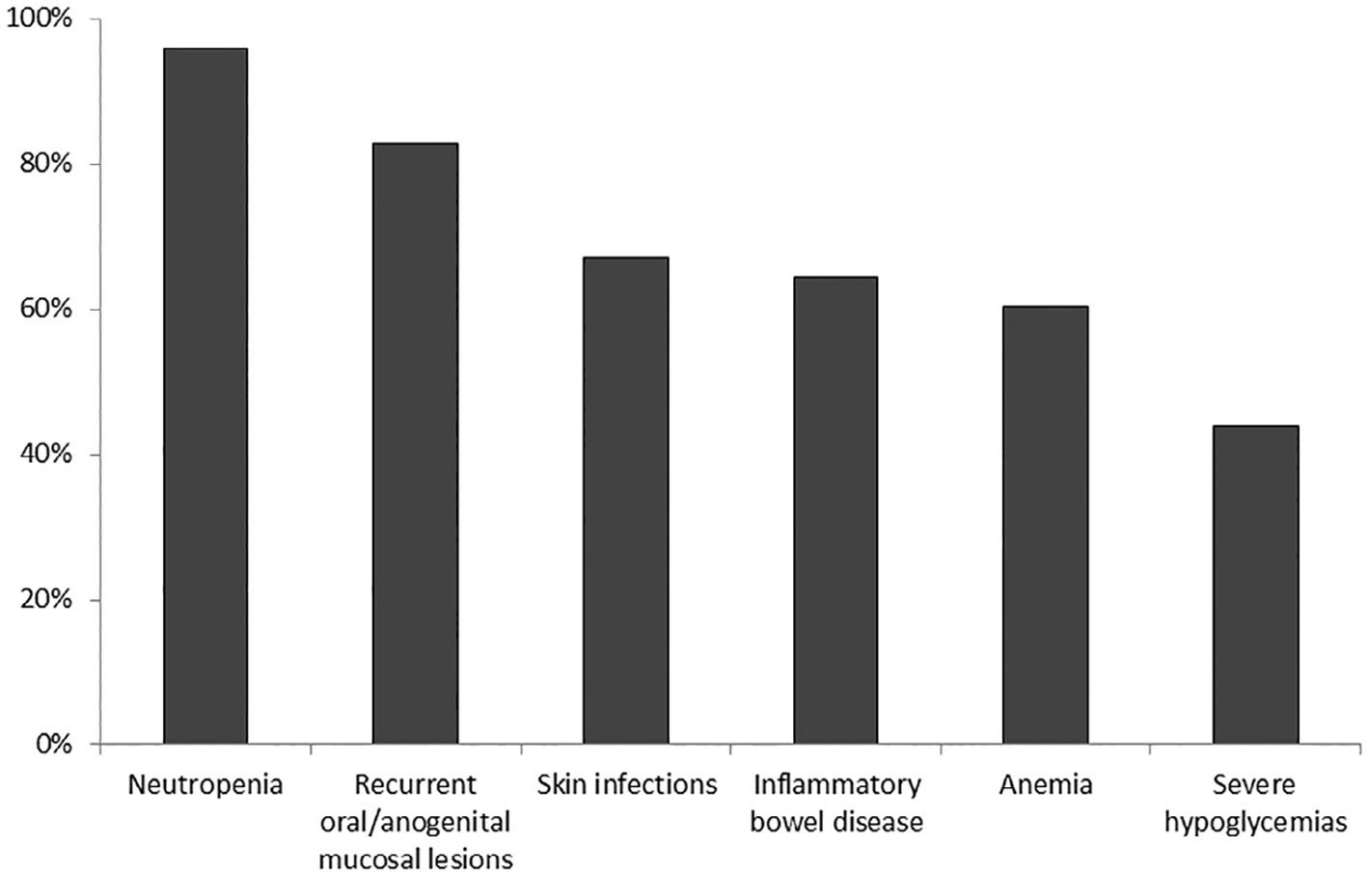
Clinical symptoms reported by GSD Ib patients/caregivers before start of empagliflozin treatment.

**FIGURE 2:**
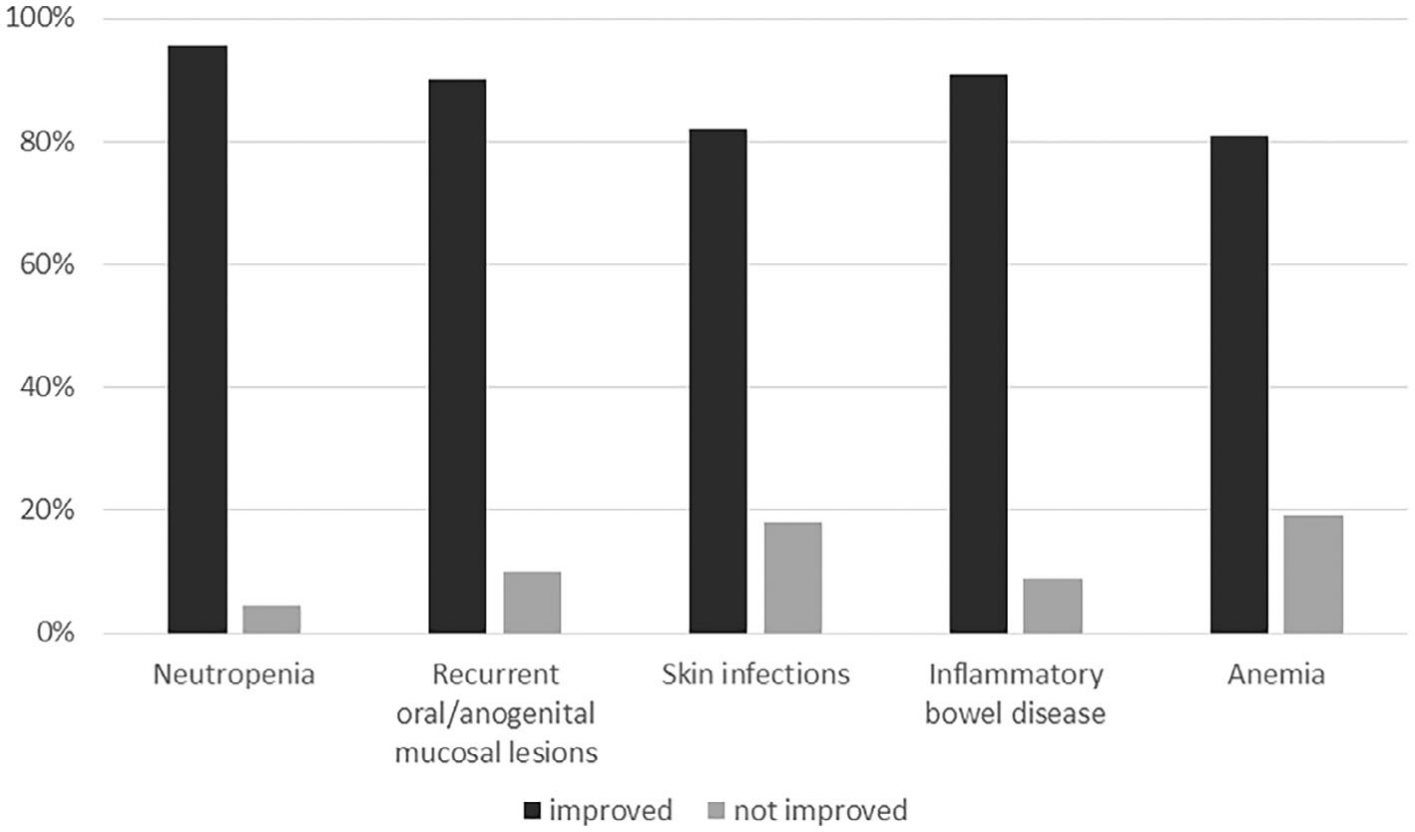
Response of neutropenia and neutropenia‐associated symptoms to empagliflozin treatment.

An overview on clinical symptoms reported by patients/caregivers before start of empagliflozin treatment is shown in Figure 1. The vast majority of patients responded very well to empagliflozin treatment with a clear amelioration of neutropenia and neutropenia‐associated symptoms (Figure 2). Neutropenia resolved in 95% of patients. Interestingly, 50% of patients reported an amelioration of severe hypoglycemias under empagliflozin treatment. Eighty‐five percent of patients (62/73) did not report difficulties in maintaining normal blood glucose levels; however, in 15% (11/73), an increase in hypoglycemias was noted compared to the time before empagliflozin treatment. Accordingly, 82% (60/73) did not need a higher amount of carbohydrates/cornstarch to maintain euglycemia. Fifteen and seven patients reported that cornstarch or Glycosade®, respectively, was tolerated under empagliflozin while it had not been tolerated before.

Empagliflozin also had a positive effect on appetite, physical performance and activity, overall well‐being of the patient, overall well‐being of the caregivers, sleep of the patient, and sleep of the caregivers (see Table 1). The number of hospitalizations was reduced in 66% of patients (48/73).

**TABLE 1:** Reported positive effects of empagliflozin treatment on different aspects of daily life of GSD Ib patients and caregivers.

**Improvement**	
Overall quality of life	82% of patients (60/73)
Appetite	63% of patients (35/56)
Physical performance and activity	75% of patients (42/56)
Overall well‐being of the patient	96% of patients (66/69)
Overall well‐being of the caregiver	94% of caregivers (50/53)
Sleep of the patient	63% of patients (33/52)
Sleep of the caregiver	67% of caregivers (28/42)
Traveling	89% of patients (65/73)
**Reduction**	
Number of hospitalizations	66% of patients (48/73)
Sick‐day leaves of patients	67% of patients (49/73)
Sick‐day leaves of caregivers of pediatric patients	27% of caregivers (12/44)

Apart from hypoglycemia, 75% of patients (55/73) experienced no side effects of the medication. Side effects reported by 18 patients included weight gain (*n* = 3), ketoacidosis (*n* = 1), urinary tract infections (*n* = 2), asymptomatic bacteriuria (*n* = 1), proteinuria (*n* = 1), dehydration (*n* = 1), increased thirst during the night (*n* = 2), genital infection (*n* = 1), constipation (*n* = 1), muscle cramps, freezing and kidney pain (*n* = 1), rash (*n* = 1), swelling of the finger joints (*n* = 1), joint pain and connective tissue inflammation (*n* = 1), dizziness and sleeping problems when taken in combination with methylphenidate (*n* = 1), and an increase in triglyceride levels (*n* = 1).

**FIGURE 3:**
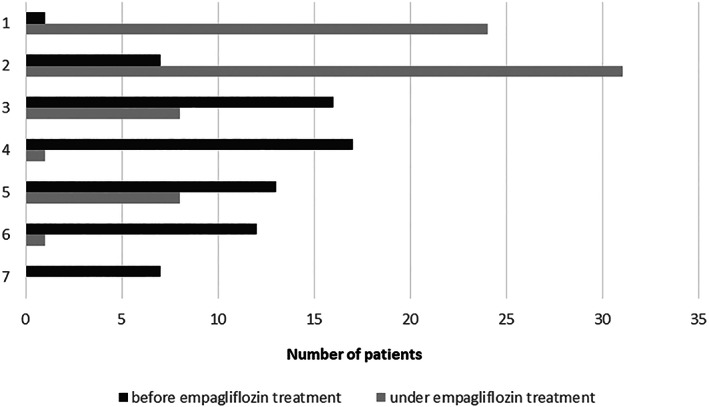
Quality of life before and after start of empagliflozin treatment ranked by patients/caregivers on a scale from 1 to 7 (1 = excellent, 7 = very poor).

Patients/caregivers were asked to rank the QoL of the patient before and after the start of empagliflozin treatment on a scale from 1 to 7 (1 = excellent, 7 = very poor). The results are displayed in Figure 3. Eighty‐two percent of patients (60/73) perceived an improved QoL, in 10% (7/73) the QoL remained unchanged, and 8% of patients (6/73) reported a deterioration of their QoL. This is reflected by a significant change in the mean rank of QoL from 4.3 before treatment to 2.2 after start of empagliflozin therapy. The majority of patients/caregivers report that it has become easier to manage their daily life since the start of empagliflozin treatment (mean rank 2.18 on a scale from 1 = yes, very much to 7 = no, not at all). Empagliflozin also had an influence on working life and school/kindergarten attendance of patients and caregivers. Less sick‐day leaves were reported in 67% of patients (49/73) and 27% of caregivers of pediatric patients (12/44), respectively. Eighty‐nine percent of respondents (65/73) reported that traveling has become easier for them under empagliflozin treatment. The overall improvement of patients' daily life on empagliflozin treatment was rated very positively by patients and caregivers (mean rank 2.2 on a scale from 1 = very much improved to 7 = no improvement at all, range 1–7).

When asked for the biggest change after start of empagliflozin treatment, patients and caregivers mentioned—among others—better well‐being; less infections, abscesses and mucosal lesions associated with less pain; less problems with PEG tubes/possibility to implement a PEG tube; better wound‐healing; better appetite; increased fasting tolerance; increased diversity of foods; less diarrhea and problems with inflammatory bowel disease; the fact that injections (G‐CSF) are no longer necessary; fewer hospital visits; weight gain; improvement of daily life; better level of activity/energy and more strength in everyday functioning; easier travel conditions; gain in autonomy and self‐confidence; and better QoL.

## 4 DISCUSSION

SGLT2 inhibitors have emerged as a new and very promising treatment option for the neutropenia‐/neutrophil dysfunction‐associated signs and symptoms in patients with GSD Ib. In contrast to G‐CSF, SGLT2 inhibitors do not only increase the neutrophil number but also improve neutrophil function.^3^, ^5^, ^6^ Experiences on more than 120 GSD Ib patients have been published within the last 2 years.^3^, ^4^, ^5^, ^6^, ^7^, ^8^, ^9^, ^10^, ^11^, ^12^, ^13^, ^14^ This study corroborates the clear improvement of neutropenia, mucosal lesions, skin infections, inflammatory bowel disease and anemia as it was reported by more than 80% of patients. Of note, many of the patients will have been included in our previous publication. Almost half of the patients could stop G‐CSF treatment, and another 42% could either reduce the dose, the frequency of dosing or both.

Daily injections may be associated with both pain and fear, especially in younger children, and this has been a major concern for many caregivers. Therefore, the fact that injections were no longer necessary was usually perceived as a significant relief.

Empaglifozin might well be superior to G‐CSF with respect to side effects and long‐term complications as SGLT2 inhibitors have not been reported to be associated with monoclonal malignancies, which have been described in several cases after long‐term treatment with G‐CSF.^15^, ^16^, ^17^, ^18^

Three quarters of patients did not experience any adverse effects, and the reported side effects in the remainder were usually not severe. As empagliflozin leads to glucosuria, GSD Ib patients may be prone to hypoglycemia under SGLT2 inhibition. This has indeed been the most common side effect observed in the cohort of 112 GSD Ib patients reported by Grünert et al. with 18% of patients affected.^4^ In accordance, an increase in hypoglycemias was noted in 15% of patients in this study, again with the notion that many patients will have been included in both studies. However, for the majority of patients maintaining normal blood glucose levels was not a problem, and the carbohydrate demand did usually not increase under empagliflozin. It can be hypothesized that this is at least partially explained by enhanced bowel health resulting in improved absorption of carbohydrates.

Empagliflozin as an oral medication also has advantages in the practical handling compared to G‐CSF injections. In contrast to G‐CSF, it does not have to be stored cooled. The cold chain can create troubles due to electricity shut downs, and cooling is also difficult while traveling. Many patients therefore reported that traveling has become much easier and requires less logistics with empagliflozin, which has a major impact on patients' mobility and QoL. Additionally, access to empagliflozin is generally easier, as in some countries, G‐CSF is only available via hospital pharmacies.

While previous studies have mainly focused on clinical response data, this study is the first to look at the reality of patients' lives under this new therapy. Our data show that patients benefit from empagliflozin therapy in many aspects of their daily lives. These include work and school life, social life and traveling. Improvements in overall well‐being, sleep, level of activity and physical strength, reduction of IBD symptoms and fewer hospital admissions allow patients to participate easier and more often in age‐adequate daily life activities and enhance a gain in autonomy and self‐confidence. From the perspective of patients and caregivers, these treatment effects may be much more significant and convincing than “hard facts” such as normalization of granulocyte counts.

Our study has several limitations. First, the questionnaire used in this survey has not been validated. However, apart from feasibility, we chose this approach to address specific aspects of living with GSD Ib, which are not sufficiently covered in commonly used validated questionnaires for the assessment of QoL. Second, the questionnaire was only available in English, which might have prevented non‐English speaking patients from participating. Nevertheless, we were able to collect data from 17 countries on 6 continents. Third, the treatment response may be overestimated, as patients with a positive treatment response may have been more motivated to complete the questionnaire.

Although empagliflozin seems to have clear benefits for patients with GSD Ib, many open questions remain. These include (1) indications for the start of treatment (presymptomatic vs. symptomatic); (2) the optimal dose and criteria for dose adaptions; (3) when to taper/stop G‐CSF; (4) optimal monitoring; (5) risk of side effects; (6) possible long‐term effects including improvement of renal function/prevention of renal insufficiency; and (7) individual pharmacogenetic and pharmacokinetic factors that influence the treatment effect. Studies on the safety and efficacy of empagliflozin are currently running in different countries (NCT04930627, phase 2 study in Poland; NCT04986735, prospective cohort study in Hong Kong). Additionally, an expert group has recently been formed to address the above‐mentioned questions and formulate treatment recommendations based on the current knowledge and scientific evidence.

In conclusion, our study on the impact of empagliflozin treatment on the daily life of patients has shown that the SGLT2 inhibitor has been a “life changer” for both patients and caregivers. By reducing neutropenia and neutropenia‐associated symptoms, empagliflozin significantly ameliorates patients' QoL and makes everyday life easier for many GSD Ib patients. There is an increasing number of instruments to quantify QoL, patient‐reported outcome measures (PROMs) and patient‐reported experience measures (PREMs) for GSD patients.^19^, ^20^, ^21^ The selection of instruments that matter most to persons with GSD will be an important next step toward a standard set of PROMs, not only for highly specialized healthcare but also for future clinical trials.

## AUTHOR CONTRIBUTIONS

Sarah C. Grünert, Saskia B. Wortmann, and Terry G. J. Derks designed the study. Sarah C. Grünert, Saskia B. Wortmann, Terry G. J. Derks, Jamas LaFreniere and Enrique Contreras were involved in the development of the questionnaire. Annieke Venema programmed the web‐based questionnaire. Jamas LaFreniere and Enrique Contreras recruited patients via the respective patient organizations. Sarah C. Grünert drafted the manuscript and all figures and tables. All other authors critically revised the manuscript and approved the final version of the manuscript.

## FUNDING INFORMATION

Not applicable.

## CONFLICT OF INTEREST STATEMENT

The authors declare that they have no competing interests concerning the content of this manuscript.

## ETHICS STATEMENT

Not applicable.

## Supporting information


**Data S1.** Supporting Information

## Data Availability

The data that support the findings of this study are available from the corresponding author upon reasonable request.
