# Supplementary material for: Patient‐reported outcomes on empagliflozin treatment in glycogen storage disease type Ib: An international questionnaire study
Source: JIMD Rep. 2023 Apr 19;64(3):252–8. doi: 10.1002/jmd2.12364 (PMC10159866; doi:10.1002/jmd2.12364)
Supplement: Supplementary file 1 — Data S1. Supporting Information [file JMD2-64-252-s001.pdf]

Empagliflozin for the neutropenia and neutrophil dysfunction of patients with glycogen storage disease type Ib.

Questionnaire about empagliflozin

Empagliflozin is a new treatment option for the neutropenia and neutrophil dysfunction of patients with glycogen storage disease type Ib.

While medical data on the efficacy and safety of empagliflozin have already been collected via a questionnaire for doctors, we are now interested to learn **what this new treatment means for patients and their families in their daily lifes.**

If you or your child is on empagliflozin treatment, please support us by filling in this questionnaire that has been designed for patients and caregivers with the help of patients and patient representatives. **Please make sure that only one questionnaire is filled in per patient.** We are planning an international webinar for patients and family members to inform them about the results of this questionnaire.

The questionnaire consists of 33 questions and will take you about **10-15 minutes.**

Thank you for your support!

Yours, sincerely,

Annieke Venema, MD-PhD candidate, University Medical Center Groningen
Dr. Terry Derks, metabolic pediatrician, University Medical Center Groningen
Prof. Sarah Grünert, metabolic pediatrician, University Children's Hospital Freiburg
Prof. Saskia Wortmann, metabolic physician, University Children's Hospital Salzburg
Enrique Lande Contreras, Patient Advocate, CureGSD1b Research Alliance www.curegsd1b.org and Spanish Association of GSD patients

* 1. I am a

- ◯ GSD Ib patient myself
- ◯ mother of a GSD Ib patient
- ◯ father of a GSD Ib patient
- ◯ other (please detail)

* 2. What is your country of residence?

* 3. What is the current age of the patient (you or your child) in years?

* 4. What was the patient's (your/your child’s) age (in years) at the start of treatment with empagliflozin?

* 5. For how many months has the patient (you or your child) taken empagliflozin now?

* 6. What is the current weight (in kg) of the patient (you or your child)?

* 7. What is the current empagliflozin dose in mg/day?

* 8. Empagliflozin is given in...

- ◯ 1 single dose per day
- ◯ split in 2 doses per day

* 9. Is empagliflozin reimbursed in your country?

- ◯ yes

- ◯ no, I have to pay myself
- ◯ other (please detail)

10. If not, does that pose a financial burden on you/your family?

- ◯ yes
- ◯ no

* 11. Has the patient (you or your child) received G-CSF treatment before the start of empagliflozin treatment?

- ◯ yes
- ◯ no

12. Was/is G-CSF reimbursed?

- ◯ yes
- ◯ no, I have to pay myself
- ◯ other (please detail)

13. Could G-CSF be stopped after the start of empagliflozin treatment?

- [ ] yes, completely stopped
- [ ] yes, but G-CSF is still given in certain situations (infections etc.)
- [ ] no, but the G-CSF dose (expressed as mg/kg/day) could be reduced
- [ ] no, but the G-CSF injection frequency could be reduced
- [ ] no, G-CSF still given at the same dose (expressed as mg/kg/day) and injection frequency as before
- [ ] no, the G-CSF dose (expressed as mg/kg/day) had to be increased
- [ ] no, the G-CSF injection frequency had to be increased

14. How difficult were regular G-CSF injections for the patient (you/ your child)?

1= very difficult

7= not difficult at all

15. If you could already stop G-CSF or reduce the dose, how do you rate the fact that injections are no longer or less often needed?

1= great relief 7= no relief

* 16. What symptoms were present BEFORE the start of empagliflozin treatment?

	yes	no	I don't know
Neutropenia	○	○	○
Recurrent oral/anogenital mucosal lesions	○	○	○
Skin infections	○	○	○
Inflammatory bowel disease	○	○	○
Anemia	○	○	○
Severe hypoglycemias	○	○	○

Other (please detail)

* 17. Which symptoms IMPROVED under empagliflozin?

	yes	no	not applicable, as symptom was not present before empagliflozin	I don't know
Neutropenia	○	○	○	○

	yes	no	not applicable, as symptom was not present before empagliflozin	I don't know
Recurrent oral/anogenital mucosal lesions	○	○	○	○
Skin infections	○	○	○	○
Inflammatory bowel disease	○	○	○	○
Anemia	○	○	○	○
Severe hypoglycemias	○	○	○	○

Other (please detail)

* 18. Have you or your child observed a positive change of....under empagliflozin treatment?

	yes	no	not applicable
Appetite of the patient	○	○	○
Physical performance/activity of the patient	○	○	○
Overall well-being of the patient	○	○	○

	yes	no	not applicable
Overall well-being of the caregiver	◯	◯	◯
Sleep of the patient	◯	◯	◯
Sleep of the caregiver	◯	◯	◯

* 19. Did the patient (you/your child) have less hospitalisations since the start of empagliflozin compared to before empagliflozin?

◯ yes

◯ no

comments

* 20. Does the patient (you/your child) have more hypoglycemias/ problems to maintain normal blood glucose concentrations under empagliflozin (compared to before empagliflozin treatment)?

◯ yes

◯ no

* 21. Does the patient (you/your child) need a higher amount of carbohydrates/cornstarch since the start of empagliflozin treatment?

◯ yes

◯ no

* 22. Did the patient (you/your child) experience any other problems/ side effects of empagliflozin?

◯ no

◯ yes (please detail):

* 23. Does the patient (you/your child tolerate cornstarch or Glycosade® during empagliflozin treatment while it was not tolerated before?

	yes	no	not applicable, cannot answer
cornstarch	◯	◯	◯
Glycosade®	◯	◯	◯

* 24. Please rate the patients (your/your child's) overall quality of life BEFORE start of empagliflozin treatment:

1= excellent 7= very poor

* 25. Please rate the patients (your/your child's) overall quality of life AFTER start of empagliflozin

treatment:

1= excellent 7= very poor

* 26. Has it become easier for the patient (you/your child) to manage your daily life since the start of empagliflozin treatment?

1= yes, very much 7= no, not at all

* 27. What is your opinion about the following topics; is it relevant (agree) or not (disagree) to your situation?

	agree	disagree	not applicable, cannot answer
Access to empagliflozin is generally/normally easier (in any pharmacy) versus GCSF (only in hospital pharmacy)	○	○	○
The G-CSF brand is normally imposed since there is no choice for patients/families	○	○	○
Some G-CSF brands are more	○	○	○

	agree	disagree	not applicable, cannot answer
simple to prepare/inject than others			
The cold chain can create troubles due to electricity shut down	◯	◯	◯
The cold chain can create troubles during traveling, due to carrying cooler boxes, etc.	◯	◯	◯

* 28. Did empagliflozin have an influence on the number of sick-leave days in kindergarten/school/at work?

- ☐ yes, LESS sick-leave days on empagliflozin than before of the patient
- ☐ yes, MORE sick-leave days on empagliflozin than before of the patient
- ☐ yes, LESS sick-leave days on empagliflozin than before of the caregiver
- ☐ yes, MORE sick-leave days on empagliflozin than before of the caregiver
- ☐ no, of the patient
- ☐ no, of the caregiver

* 29. Has travelling become easier for the patient (you/your child) under empagliflozin?

- ◯ yes
- ◯ no

* 30. How would you rate the improvement of the patients (your/your child's) daily life after start of empagliflozin treatment?

1= very much improved — 7= no improvement at all

31. What is the patients (your /your child's) biggest change after start of empagliflozin treatment?

32. Do you have remarks or concerns about the treatment with empagliflozin?

33. Is there anything else you would like to let us know?

Thank you for your support!

Done

Powered by
SurveyMonkey®
See how easy it is to create a survey.

Privacy & Cookie Notice
